# Is there a cognitive advantage in inhibition and switching for bilingual children? A systematic review

**DOI:** 10.3389/fpsyg.2023.1191816

**Published:** 2023-06-16

**Authors:** Niels Planckaert, Wouter Duyck, Evy Woumans

**Affiliations:** ^1^Department of Translation, Interpreting, and Communication, Ghent University, Ghent, Belgium; ^2^Department of Experimental Psychology, Ghent University, Ghent, Belgium; ^3^The Accreditation Organisation of the Netherlands and Flanders (NVAO), Den Haag, Netherlands

**Keywords:** bilingual advantage, executive functioning, cognitive development, bilingualism, response inhibition, interference suppression, switching

## Abstract

Several studies have pointed to beneficial effects of bilingualism on executive functioning. However, observations of these beneficial effects have at times proven difficult to reproduce. Moreover, findings of studies on cognitive effects of bilingualism have been contested altogether. These contradictory outcomes leave the research field of bilingualism at unease. In the present review article, we aim to give a systematic overview of previous research on bilingual advantages in inhibition and switching in children up to the age of 12. Particular attention is paid to the experimental tasks that have been applied and the persistence of possible effects throughout critical and post-critical periods for cognitive development in children. In doing so, the review gives an insight in both the validity and robustness of possible domain-general cognitive effects of bilingualism in children. Terminological issues are also discussed.

## Introduction

For quite some time now, research has been conducted on the cognitive impact of bilingualism in young children. In early work on bilingualism, the acquisition of languages other than the native one was considered a risk factor for verbal and non-verbal cognitive development. Studies reported that bilinguals performed worse relative to their monolingual peers on a variety of variables, ranging from smaller vocabulary sizes (Smith, 1949) to impaired general intelligence (Darcy, 1946). However, in a seminal paper, Peal and Lambert (1962) reported that 10-year-old bilinguals actually outperformed their monolingual peers on tests of intellectual reasoning. The result was later confirmed by Ben-Zeev (1977) for 5- to 8-year-olds, and, in fact, a bulk of research spread over the past 20 years has actually shown that bilingualism might foster cognition rather than impede it. As such, in a longitudinal study with 5-year-olds, Woumans et al. (2016) found that only bilinguals improved significantly on intelligence over a period of one year. An extensive branch of research has focused on the effect of bilingualism on executive functioning (EF), which is to be understood as covering a broad range of cognitive functions that are used to control and regulate actions and thought. Around the turning of the centuries, a consensus was reached that a bilingual's languages are always simultaneously active and interacting (Hermans et al., 1998; Costa et al., 2000; Duyck, 2005; Brysbaert and Duyck, 2010), resulting in constant cognitive conflict for the bilingual (Green, 1998; Woumans et al., 2016). It became a common research question whether this conflict, as a kind of cognitive exercise, has repercussions outside of the verbal domain. Because language control, and therefore resolution of language conflict, relies on executive functions, this has gradually led to an almost exclusive focus on advanced executive functioning as a by-product of bilingualism, moving away from the former focus on general intelligence.

Several studies have pointed to beneficial effects of bilingualism on executive functioning in children. To illustrate, Kovács and Mehler (2009) revealed through three eye-tracking studies with 7-month-old infants that they outperformed matched monolinguals on cognitive control abilities. Kang and Lust (2019) found that bilingual language proficiency in 8-year-old children was a predictor for their EF performance. Tran et al. (2019) detected a similar bilingualism effect on cognitive control processes measuring selective attention, switching, and inhibition in a longitudinal study with 3- to 4-year-olds. In a large-scale study (*N* = 18,200) with children aged 5 to 7, bilingualism was found to moderate the effects of socio-economic status (SES) by ameliorating the detrimental consequences of low-SES on EF (Hartanto et al., 2019).

These beneficial effects have, however, proven difficult to systematically reproduce, resulting in an ongoing profound debate on the existence and scope of the bilingual advantage. Both for EF and for intelligence, very diverging results have been reported. As such, Dick et al. (2019) found no evidence for a bilingual executive control advantage in a large sample (*N* = 4,524) of 9- to 10-year-olds who were tested for inhibitory control, attention, task switching, and cognitive flexibility. Similar results were obtained in a study by Jaekel et al. (2019) on bilingual Turkish immigrant children aged 5 to 15 years. Equally, no effect of bilingualism on tasks of inhibition, updating, and shifting, i.e., components of EF, were found in a study by Arizmendi et al. (2018) among 7- to 9-year-olds. A meta-analysis by Gunnerud et al. (2020) targeting children aged 18 and under gave little support for a bilingual advantage in overall EF.

Those outcomes are in line with what has been identified in bilingual advantage literature targeting adult populations. In a recent large-scale study with 11,000 participants, bilinguals showed no advantage over monolinguals in a battery of 12 EF tasks (Nichols et al., 2020). In fact, findings on bilingual cognitive advantage effects have been contested altogether, starting with the hallmark criticism study of Paap et al. (2015). Issues that have been raised in studies skeptical of bilingual advantages include the observation of and publication bias for frequent null results, insufficient sample sizes, and the use of questionable research methods. Or, it is claimed that participants would be inadequately matched on background variables and any significant differences in performance may well reflect task-specific mechanisms instead of domain-free executive functioning abilities (Paap et al., 2015; Laine and Lehtonen, 2018). The criticism was further corroborated by a meta-analysis indicating no systematic support for the benefits in cognitive control functions associated with bilingualism (Lehtonen et al., 2018). Nevertheless, the meta-analysis by De Bruin et al. (2015) showed a significant bilingual advantage effect across published studies, although the simultaneously observed publication bias for bilingual advantages received more attention and fueled the doubt about the bilingual advantage. Moreover, in his own meta-analysis, Grundy (2020) argued “that there are several reasons, often overlooked, that lead to failed replications, and that when group differences do appear on EF tasks, despite these issues, performance favors bilinguals far more often than monolinguals” (p. 177), supporting his claim with Bayesian analysis of 167 independent studies that resulted in a Bayes Factor of BF10 = 2.91 × 10^8^, classified as “decisive” evidence.

Taken altogether, the strongest evidence for a bilingual advantage seems to come from studies targeting very young children and aging adults (Bialystok et al., 2005; Woumans et al., 2015), suggesting that bilingualism mainly impacts the sensitive periods of cognitive development and cognitive decline. Development of the cognitive control (CC) system is one the most essential processes in childhood (Diamond, 2002), evolving rapidly, especially between the ages of three to six (Best and Miller, 2010). Beneficial effects of bilingualism are reported in children from birth up to the age of six (Martin-Rhee and Bialystok, 2008; Kovács and Mehler, 2009; Morales et al., 2013; Crivello et al., 2016), but it appears that null effects arise more frequently in children over the age of six (Martin-Rhee and Bialystok, 2008; Abdelgafar and Moawad, 2015). As such, it may be that the acquisition of a second language (L2) in addition to acquisition of the first (L1) accelerates cognitive development during the critical period, but that afterwards the monolinguals catch up again with their bilingual peers. Likewise, studies into cognitive decline and its relation to bilingualism tend to show a temporary bilingual advantage (e.g., Abutalebi et al., 2015). As such, the onset of dementia, for instance, is found to be delayed by ~4 to 5.5 years in bilinguals (Craik et al., 2010; Alladi et al., 2013; Woumans et al., 2015). This temporary nature of differences is why, in the present review focusing on children, we have differentiated between studies targeting children younger than 6 years old (critical) and studies targeting children between the ages of 6 and 12 (post-critical). In doing so, we aimed to give an overview of previous research reporting on bilingual advantage effects in young children, shedding light on the validity and robustness of possible cognitive effects of L2 acquisition. Specifically, this review considers research on inhibition and switching, two constructs which are frequently related to bilingualism as the concept of speaking two languages in itself requires inhibition of the non-spoken language and switching between languages. Particular attention is paid to the experimental tasks that have been applied and the persistence of possible effects throughout critical and post-critical periods for cognitive development.

## Method

All articles considered in our analysis were retrieved from Web of Science through two systematic searches. The searches “((ALL = (bilingual advantage)) AND ALL = (children)) AND ALL = (executive control)” and “((ALL = (immersion)) AND ALL = (cognitive control)) AND ALL = (children)” resulted in 281 and 49 hits, respectively. All hits were automatically filtered on “Document type: Article” and “Web of Science categories: Psychology Experimental or Linguistics,” reducing the total number of hits to 189. During manual filtering, articles targeting age groups older than 12 were excluded and the sample was limited to articles looking into the constructs of switching and inhibition. Articles with a focus that did not entail a direct comparison between bilinguals or second language learners, and monolinguals were also removed, resulting in a final sample of 58 references.

From each article, age range, number of participants, targeted measures and tasks applied were extracted. It was indicated for each task if a significant bilingual advantage was detected. The results of this process are summarized in Table 1. In the naming of the measures and tasks, the terminology applied by the original author was kept. Articles were visually split up in the table according to the age range they were reporting on, in an attempt to visualize the persistence of any possible effects through the critical and post-critical period for cognitive development. The threshold for the transition from critical to post-critical was set at the age of 6, as the cognitive control system is especially evolving rapidly between the ages of three and six (Best and Miller, 2010).

**Table 1:** Overview of studies on switching and inhibition in critical and post-critical age groups.

**Reference**	**Age group**	**# participants**	**Languages**	**Measures**	**Tasks**	**Advantage?**
**Critical age group**						
Baralt and Mahoney (2020)	4 to 7	*N* = 35 Mono = 20 Bi = 15	English Spanish	Interference suppression Response inhibition	Simon Task Flanker Task	No Yes
Beaudin and Poulin-Dubois (2022)	1;8 to 2;3	*N* = 81 Mono = 39 Multi = 42	English French Other	Response inhibition	Early Executive Functions Questionnaire	Yes
Bialystok et al. (2010)	3 to 4;6	*N* = 162 Mono = 106 Bi = 56	English French	Response inhibition Task switching Inhibitory control Shifting	Luria's Tapping Task Opposite Worlds Task ANT Flanker task Reverse Categorization	Yes Yes No Yes
Castillo et al. (2022)	5 to 7	*N* = 7,846 Mono = 7,095 Bi = 522 SLL = 229	English Non-English	Cognitive flexibility Inhibitory control	DCCS Task Teacher report	Yes Yes, except SLL
Cho et al. (2021)	3;5 to 5;5	*N* = 99 Mono English = 34 Mono Korean = 33 Bi = 32	English Korean	Inhibitory control	Stroop Task	Yes, but only for Mono English
Crivello et al. (2016)	1;10 to 2;8	*N* = 82 Mono = 43 Bi = 39	English French	Conflict inhibition Response inhibition Response control	Reverse Categorization Task Shape Stroop Task Gift Delay Task Multilocation Task	Yes No No
Diaz and Farrar (2018)	3;4 to 5;5	*N* = 65 Mono = 33 Bi = 32	English Spanish	Inhibitory control Cognitive flexibility	Day/night Stroop-like Task DCCS Task Bear/Dragon Simon Says Task	Yes (all considered as one)
Esposito et al. (2013)	3;1 to 6;3	*N* = 51 Mono = 25 Bi = 26	English Spanish	Response inhibition Interference suppression	Day/Night Task Bivalent Shape Task	No Yes
Foy and Mann (2014)	5	*N* = 60 Mono = 30 Bi = 30	English Spanish	Interference suppression	Verbal auditory Go/No-go Task Non-verbal auditory Go/No-go Task	No Yes
Grote et al. (2021)	4	*N* = 60 Mono = 40 Bi = 20	English Spanish	Response inhibition	Day/Night Task	Yes
Kalashnikova and Mattock (2014)	3 to 5	*N* = 66 Mono = 33 SLL = 33	English Welsh	Attentional control	DCCS Task	Yes
Martin-Rhee and Bialystok (2008)—Study 1	4 to 5	*N* = 34 Mono = 17 Bi = 17	English French	Interference suppression	Simon Task: immediate Simon Task: short delay Simon Task: long delay	Yes No No
Martin-Rhee and Bialystok (2008)—Study 2	4	*N* = 41 Mono = 20 Bi = 21	Chinese English French Spanish	Interference suppression Response inhibition	Simon Task Stroop Picture Naming Task (Day/Night, Cat/Dog)	Yes No
Nayak and Tarullo (2020)	3;6 to 4;6	*N* = 115 Mono = 62 Bi = 53	English Non-English	Inhibitory control	Cool DCCS Task Hot DCCS Task	No Yes
Nguyen and Astington (2014)	3 to 5	*N* = 72 Mono = 48 Bi = 24	English French	Conflict inhibition	Stroop Task	No
Poulin-Dubois et al. (2011)	2	*N* = 63 Mono = 30 Bi = 63	English French	Conflict resolution Delay	Multilocation Task Shape Stroop Task Reverse Categorization Task Snack Delay Gift Delay	No Yes No No No
Poulin-Dubois et al. (2022)	1;5	*N* = 102 Mono = 60 Bi = 42	English French	Inhibitory control Shifting	Detour Reaching Task Delayed Response Task	No No
**Critical age group**						
Santillan and Khurana (2018)	4 to 6	*N* = 1,146 Mono = 733 Bi = 216 SLL = 197	English Spanish	Inhibitory control	Pencil-tapping Task	Yes
Tran et al. (2019)	3 to 5	*N* = 96 Mono = 52 Bi = 44	Cantonese English Spanish Vietnamese	Switching Response inhibition Complex motor response inhibition Simple Motor response inhibition	DCCS Task Day/Night Stroop Task Bear/Dragon Task Gift Delay Task	Yes Yes No Yes
Verhagen et al. (2020)	2	*N* = 95 Mono = 58 Bi = 37	Dutch Non-Dutch	Inhibitory control Inhibitory control and attentional shifting	Spatial conflict task Early Childhood Behavior Questionnaire	No No
Verhagen et al. (2017)	2;11 to 4;3	*N* = 1,029 Mono = 829 Bi = 200	Dutch Non-Dutch	Inhibitory control Self-control	Stroop Task Gift Delay Task Gift-in-bag Task	Yes No No
White and Greenfield (2017)	3 to 5	*N* = 303 Mono = 83 Bi = 148 SLL = 72	English Spanish	Inhibitory control Attention shifting	Spatial Conflict Arrows Go/No-go Task Silly Sounds Stroop Task Something's the Same	Yes, but only for Bi as opposed to Mono (analysis considers all tests at once)
Wimmer and Marx (2014)	3 to 5	*N* = 114 Mono = 71 Bi = 70	English Non-English	Inhibitory control in visual perception	Ambiguous figures production plus feature identification False belief task Droodle task	Yes, only for feature identification No No
Woumans et al. (2016)	5 to 6	*N* = 54 Mono = 27 SLL = 27	Dutch French	Interference suppression	Simon task	No
Yang and Yang (2016)	5 to 6	*N* = 63 Mono = 31 Bi = 32	English Korean	Attention system	ANT	Yes
Yang et al. (2011)	4	*N* = 56 Mono = 41 Bi = 15	English Korean	Executive functioning	ANT	Yes
**Post-critical age group**						
Abdelgafar and Moawad (2015)	7 to 10	*N* = 50 Mono = 25 Bi = 25	Arabic English	Response inhibition	Stroop Task	No
Antoniou et al. (2016)	4;5 to 12;2	*N* = 136 Bilectal = 64 Multi = 47 Mono = 25	English Greek	Inhibition Switching	Soccer Task, Simon Task Color-Shape Task	Yes Yes
Arizmendi et al. (2018)	7 to 9	*N* = 247 Mono = 167 Bi = 80	English Spanish	Inhibition Shifting	Classic Stroop Task, Stroop Cross-Modal Task, Stop-Signal Task Pirate Sorting Task, Global-Local Task	No No
Bialystok and Barac (2012)—Study 1	7 to 9	*N* = 100 Mono = 35 Bi = 65	English Hebrew Russian	Response inhibition Task switching	Flanker Task Blue Horse/Red Cow	Yes Yes
Bialystok and Barac (2012)—Study 2	7 to 11	*N* = 80 Bi = 80	English French	Task switching	Blue Horse/Red Cow	Yes
Bialystok and Viswanathan (2009)	8	*N* = 90 Mono = 30 Bi = 60	English Non-English Tamil/Telugu	Response suppression Inhibitory control Switching	Faces Task	No Yes Yes
Cape et al. (2021)	8;8 to 10;0	*N* = 59 Mono = 30 Bi = 29	English Gaelic	Switching Response inhibition	Test of Everyday Attention for Children: Creature Counting Walk/Don't Walk Opposite worlds	No No Yes
**Critical age group**						
Cottini et al. (2015)	8 to 10	*N* = 104 Mono = 49 Bi = 55	German Italian	Inhibitory control	Global/local Task	Yes
Crespo et al. (2019)	5 to 11	*N* = 156 Bi = 156	English Spanish	Shifting Switching Mixing cost	DCCS Task	Yes
Czapka and Festman (2021)	9	*N* = 122 Mono = 66 Multi = 56	German Non-German	Switching	Wisconsin Card Sorting Task	No
Czapka et al. (2020)	9	*N* = 168 Mono = 69 Multi = 57	German Non-German	Response inhibition Interference inhibition	Go/No-go Task Bivalent Shape Task	No No
de Abreu et al. (2014)	8	*N* = 81 Mono = 33 Bi = 33 (Bi with SLI = 15)	Portuguese Luxembourgish	Interference suppression	Flanker Task	Yes
Dick et al. (2019)	9 to 10	*N* = 4,524 Mono = 2,784 Bi = 1740	English Non-English	Inhibitory control Switching Inhibitory control	NIH Toolbox Flanker Inhibitory Control and Attention Test NIH Toolbox DCCS Task Stop-signal Task	No No No
Dunabeitia et al. (2014)	8 to 13	*N* = 504 Mono = 252 Bi = 252	Basque Spanish	Response inhibition	Classic Stroop Task Numerical Stroop Task	No No
Ebert et al. (2019)	6;0 to 8;11	*N* = 154 Mono = 64 Bi = 90	English Spanish	Attentional control	Flanker Task	No
Escobar et al. (2018)	7	*N* = 34 Mono = 17 Bi = 17	English Non-English	Inhibitory control	DCCS Task Day/Night Stroop Task	No No
Esposito (2020)	6 to 10	*N* = 288 Mono = 204 Bi = 84	English Spanish	Switching Inhibitory control Behavioral EF	Trail Making Task Bivalent Shape Task, Simon Task Behavioral Rating Inventory of Executive Functions (BRIEF)	No No Yes
Filippi et al. (2022)	7 to 15	*N* = 154 Mono = 77 Multi = 77	English Non-English	Visual interference suppression Response Inhibition	Simon Task Go/No-go Task	No No
Johann et al. (2022)	7 to 10	*N* = 228 Mono = 133 Bi = 95	German Non-German	Inhibition Shifting/flexibility	Go/No-go Task AX-continuous performance Task Cued task switching Task switching with alternating runs	No No No No
Kapa and Colombo (2013)	5;8 to 14;11	*N* = 79 Mono = 22 Bi early = 21 Bi late = 36	English Spanish	Conflict resolution	ANT	Yes
Karimi and Rad (2021)	6 to 8	*N* = 56 Mono = 28 Bi = 28	English Persian	Inhibitory control	Flanker Task	Yes
Kaushanskaya et al. (2014)	5 to 7	*N* = 38 Mono = 19 Bi = 19	English Spanish	Task shifting	DCCS Task	No
Martin-Rhee and Bialystok (2008)—Study 3	8	*N* = 32 Mono = 19 Bi = 13	English Hebrew Russian	Response inhibition Interference suppression	Univalent Arrows Task Bivalent Arrows Task (modifications of Simon)	No Yes
**Critical age group**						
Neveu et al. (2021)	8 to 10	*N* = 66 Mono = 33 SLL = 33	English Spanish	Inhibition Shifting, Switching	Flanker Task Go/No-go Task DCCS Task	No Yes, only at T1 (longitudinal study with two T's) No
Nicolay and Poncelet (2013)	8;1 to 9;1	*N* = 104 Mono = 51 Bi = 53	English French	Response inhibition Interference inhibition	“The Bat” from KITAP (Go/No-go task) ANT	No No
Papastergiou et al. (2022a)	7 to 11	*N* = 70 Mono = 38 Bi = 32	English Greek	(Inhibition, Shifting) = > add up to Technical efficiency (new concept)	Non-verbal Stroop Task Color-Shape Task	Yes (considered as one)
Papastergiou et al. (2022b)	5;3 to 9	*N* = 59 Mono English = 25 Mono Greek = 15 Bi = 19	English Greek	Inhibition Shifting	Non-verbal Stroop Task Color-Shape Task	Yes (only as opposed to Mono English) No
Park et al. (2019)	8 to 12	*N* = 84 Mono = 35 Bi = 23 (Mono with DLD = 17) (Bi with DLD = 9)	English Non-English	Attention	ANT	No
Park et al. (2022)	9 to 10	*N* = 476 Mono = 358 Bi = 118 (More or less, numbers don't add up)	English Spanish	Interference suppression Inhibitory control	Bivalent Shape Task Simon Task	Yes Yes
Poarch and van Hell (2012)—Study 1	5 to 8	*N* = 75 Mono = 20 SLL = 19 Bi = 18 Tri = 18	English German Other	Conflict resolution	Simon Task	Yes but not for SLL
Poarch and van Hell (2012)—Study 2	6 to 8	*N* = 56 SLL = 19 Bi = 19 Tri = 18	English German Other	Conflict resolution	ANT	Yes
Puric et al. (2017)	8 to 10	*N* = 58 Mono = 22 SLL high exposure = 19 SLL low exposure = 22	English German Serbian	Inhibition Shifting	Non-verbal Stroop Task Local-global Task, Color/Shape Task	No No
Ross and Melinger (2017)—Study 1	6 to 9	*N* = 147 Mono = 45 Bi = 54 Bilectal = 48	English Non-English	Inhibition	Simon task Flanker Task	Yes No
Ross and Melinger (2017)—Study 2	6 to 9	*N* = 90 Mono = 21 Bi = 49 Bilectal = 20	English Non-English	Switching	Berg Card Sorting Task	No
Simonis et al. (2020)	10	*N* = 230 Mono = 102 Bi = 128	Dutch English French	Inhibitory control, Interference suppression Switching	Simon Task, ANT DCCS Task	No No No
Zeng et al. (2019)	6 to 10	*N* = 37 Mono = 17 Bi = 20	English Non-English	Executive functioning	Simon Arrows Task	Yes

Acronyms key: Mono, Monolingual; Bi, Bilingual; SLL, Second Language Learner; Bilectal, Speaking two dialects; SLI, Specific Language Impairment: these results are not included for the analysis in the present review; DLD, Developmental Language Disorder: these results are not included for the analysis in the present review.

## Results

Both research into measures associated with inhibition and research into measures associated with switching was considered in the present review article. As could be expected, the overview includes mixed results for virtually every measure and every task at hand. However, the main aim of this analysis is to detect trends throughout these mixed results in both grouping them and discussing them individually. One way of grouping them is to consider them according to age range, as was done for the visual representation in Table 1.

Moreover, grouping was also done in interpreting the diverse terminology applied in pinpointing measures. We argue a great deal of different tasks and labels really come down to measuring two major constructs in executive functioning: inhibition and switching. Inhibition includes, among others, measures that have previously been called response inhibition, interference suppression, inhibitory control, and conflict resolution. Switching includes measures such as shifting, task switching and cognitive flexibility. Within the larger constructs of inhibition and switching, different tasks are individually discussed.

### Inhibition

Tasks that have frequently been applied for measuring this cognitive function in our sample were Stroop-like tasks (20 times), Simon-like tasks (18 times), and Flanker-like tasks (16 times). There appear to be some differences in tasks applied for children under the age of 6 and tasks applied for children between the ages of 6 and 12. Flanker-like tasks were used more often among older children. Some tasks, such as gift delay tasks and tapping tasks, were only used with younger children, while others such as stop-signal tasks and bivalent shape tasks were only used with older children. Interestingly, several authors considered the Dimensional Change Card Sort Task (DCCS) (Zelazo, 2006) and variations thereof as a measure of inhibition (Poulin-Dubois et al., 2011; Crivello et al., 2016; Diaz and Farrar, 2018; Escobar et al., 2018; Nayak and Tarullo, 2020). In the DCCS Task, participants are asked to sort cards, switching between different rules to do so. Hence, we argue this task is rather a measure of switching and do not include it in the present section on inhibition, as the DCCS protocol (Zelazo, 2006) states that the inclusion of pre- and post-switch phases requires the formulation and use of higher-order rules for selecting which pair of rules to use on any particular trial. In other words, participants must constantly switch between rules in response to the instruction given.

In the complete set of literature, a bilingual advantage for inhibition was reported 42 out of 91 times (46%). In the subset of studies on children up to 6, a bilingual advantage was detected 25 out of 45 times (56%). In the subset with older children, the advantage was reported 17 out of 46 times (37%). More details on the tasks applied and the frequencies with which they led to bilingual advantages are to be found in Table 2.

**Table 2:** Bilingual advantage detection rates for critical and post-critical age groups.

**Tasks**	**Critical**		**Post-critical**	
	**# BA studies** ^*^	**Percentage**	**# BA studies** ^*^	**Percentage**
*Inhibition*	25/45	56	17/46	37
Stroop	8/11	73	2/9	22
Simon	3/8	38	6/10	60
Flanker	3/4	75	5/12	42
Go/No-go	3/5	60	1/6	17
Gift delay	1/6	17	0/0	/
Bivalent shape	1/1	100	1/3	33
Questionnaire	3/4	75	0/0	/
Stop-signal	0/0	/	1/3	33
Tapping task	2/2	100	0/0	/
Other	1/4	25	1/3	33
*Switching*	10/16	63	8/24	33
DCCS—Color/shape	7/9	78	5/15	33
Global/local	0/0	/	1/3	33
Opposite worlds	1/1	100	1/1	100
Multilocation	0/2	0	0/0	/
Questionnaire	1/2	50	0/0	/
Other	1/2	50	1/5	20

* BA stands for “Bilingual advantage detected in X/X cases.”

Delay-type tasks (where response is delayed, such as gift delay) were administered 6 times, all in studies targeting children younger than 6. An effect on this task was detected only once (1/6).

Interestingly, in 4 studies questionnaire-like methods were applied to tap into inhibition, with either teachers or parents responding to the surveys (Esposito, 2020; Verhagen et al., 2020; Beaudin and Poulin-Dubois, 2022; Castillo et al., 2022) instead of the more common experimental tasks. In those questionnaires, parents or teachers are asked about a child's behavior. Questions may for example include “How often in the past two weeks did your child follow a simple instruction for a task that they were interested in (e.g., getting a nearby toy), without getting distracted” (Hendry and Holmboe, 2021). Although questionnaires might be more susceptible to biases, they might also provide a more comprehensive overview of the participants' behavior and functioning. In 3 out of 4 studies, the questionnaires pointed toward a bilingual advantage in executive functioning. It remains unclear why an advantage was not detected in Verhagen et al. (2020) while it was detected in Beaudin and Poulin-Dubois (2022), as both studies targeted similar age groups and had a comparable number of participants (95 and 81, respectively). However, Verhagen et al. (2020) applied the Early Childhood Behavior Questionnaire (ECBQ), originally designed by Putnam et al. (2006) to assess attentional focusing, inhibitory control, and attentional shifting. This use of the ECBQ was criticized by Hendry and Holmboe (2021), as they argue the questionnaire was originally developed to assess a range of temperament traits and these are not synonymous to executive functioning abilities, although some of them are closely related. In line with this criticism, Hendry and Holmboe (2021) developed the Early Executive Functions Questionnaire (EEFQ), notably the questionnaire that was used in the study by Beaudin and Poulin-Dubois (2022). Hence, it should be noted that the bilingual advantage effect was detected when using a questionnaire that was designed to target EF and was not detected in the ECBQ.

#### Flanker-like tasks

Flanker-like tasks include the standard Flanker Task (Eriksen and Eriksen, 1974) as well as the Attention Network Task (ANT) (Fan et al., 2002), which is a combination of the cued reaction time (Posner, 1980) and the Flanker Task. Flanker tasks usually involve five arrows pointing to different directions, the participant having to indicate the direction the middle arrow points toward. This type of task led to a detected bilingual advantage in half of the occasions (8/16). Interestingly, the advantage was detected 3 out of 4 times with participants younger than 6 years old, whereas it was only detected 5 out of 12 times with older children, indicating more variation in studies targeting older children.

#### Stroop-like tasks

Stroop-like tasks were performed 20 times throughout our sample and include tests denominated as Stroop Task (Stroop, 1935) and variations thereof, and the child version Day/Night Task (Gerstadt et al., 1994). The Stroop Task involves both congruent and incongruent trials in which participants have to say the color of a word presented (e.g., the word “blue” is displayed in green). In younger children, this type of task led to a significant effect 8 times out of 11. In older children, a bilingual advantage was only identified in 2 out of 9 instances.

#### Simon-like tasks

The Simon Task (Simon and Rudell, 1967) was applied 18 times in our sample, of which it led to a significant effect in 9 instances (50%). In the Simon Task, stimuli are presented both left and right on the screen, and participants are asked to respond according to the stimuli's color (e.g., left for red, right for blue). The task involves both congruent and incongruent trials. In younger children a bilingual advantage was detected 3 out of 8 times, whereas in older children it was detected 6 out of 10 times.

#### Go/No-go tasks

The Go/No-go Task (Donders, 1969) and its variation Bear/Dragon Task (Jones et al., 2003) were administered 11 times. In the Go/No-go Task, participants' ability to withhold a response is measured. According to the instruction given, they should press or not press a button. In the younger age group, significant effects were found in 3 out of 5 instances, which is considerably more than in the older age group (1/5).

### Switching

Measures such as shifting, task switching, and cognitive flexibility were considered to relate to the construct of switching. Moreover, we identify DCCS-like tasks as measuring switching, whereas other authors have applied these for measuring inhibition (cf. supra). In fact, the DCCS Task (Zelazo, 2006) and its variations, including the Color/Shape Task (Miyake et al., 2004), are by far the most applied tasks for measuring switching in our set of studies (used 24/40 times). Other tasks include the Opposite Worlds Task (Manly et al., 2001), the Multilocation Task (Zelazo et al., 1998), and the Global/Local Task (Navon, 1977).

Across all studies, a bilingual advantage for switching was reported 18 times (18/40; 45%). In the subset of studies on children onto 6, a bilingual advantage was detected 10 out of 16 times (63%), whereas in the subset with older children the advantage was reported 8 out of 24 times (33%). More details on the tasks applied and their respective bilingual advantage detection rates are to be found in Table 2.

The Opposite Worlds Task (Manly et al., 2001) was administered twice and resulted in a bilingual advantage effect on both occasions, both in 3- to 4-year-olds and in 8- to 10-year-olds. This task requires to switch between naming systems; naming animals first by their true names (e.g., “cow”) and later by their silly name (e.g., “pig” for cow). The Multilocation Task (Zelazo et al., 1998) was likewise performed twice, on both occasions in children younger than 6. The task involves objects being hidden in different locations, children having to respond to instructions to try and find them. No bilingual advantage was detected.

#### DCCS-like tasks

DCCS-like tasks include the standard DCCS Task (Zelazo, 2006), the Color/Shape Task (Miyake et al., 2004), the Blue Horse/Red Cow Task (Barac and Bialystok, 2012) and the Reverse Categorization Task (Carlson et al., 2004). This type of task led to a bilingual advantage being identified in half of the occasions (12/24). The advantage was detected relatively more often in younger children (7/9) than in older children (5/15).

## Discussion

Quite a lot of research has been conducted on the effect of bilingualism on inhibition and switching in children. Although findings of bilingual advantages on such cognitive control measures have dominated the research field for quite some time now, a debate is still raging on when, how, why, and even if these advantages appear. The present review set out to distinguish between bilinguals pre and post the critical age of development in order to determine whether age is a possible modulator of the effect. Our review included 58 articles on the topic and covered a total of 125 tasks.

It appears that in general, more research has been conducted on the construct of inhibition (90 tasks) than on the construct of switching (40). One possible explanation for this apparent focus of research is that all bilinguals constantly need the ability to inhibit input from the non-used language, while not all bilinguals have to switch very often (e.g., when the use of either language is restricted to different contexts). It should be noted, however, that inhibition was also defined as a broader construct than switching, including both response inhibition and interference suppression. Overall, a great deal of variation was present in the outcomes of these tasks, which strongly relates to the criticism uttered on frequent null results and the failure to reproduce bilingual effects (Paap et al., 2015). Moreover, variation in the current review was also omnipresent in terminology applied by different authors. Measures of inhibition were, among others, called “response inhibition,” “inhibitory control,” “conflict inhibition,” and “conflict resolution,” without defining resemblances and differences between any of those concepts. There appeared to be no consensus on which measures EF consists of exactly, nor what tasks can be used for measuring them, as was previously also indicated by among others Morra et al. (2018). Simon tasks (Simon and Rudell, 1967), for example, were applied to evaluate interference suppression (Baralt and Mahoney, 2020) as well as conflict resolution (Poarch and van Hell, 2012). The DCCS task was used to tap into switching (Simonis et al., 2020), inhibitory control (Escobar et al., 2018), and attentional control (Kalashnikova and Mattock, 2014). In considering the somewhat broader concepts of inhibition and switching, we tried to accommodate for these issues. As for different tasks applied to different age ranges, measures of Stroop, Simon, and Flanker were all administered among children of both critical and post-critical age, whereas parent/teacher questionnaires, gift delay tasks, and tapping tasks were employed solely among children under the age of 6 and stop-signal tasks only among older children.

Looking at tasks of inhibition, it was noted that Stroop- and Flanker-like tasks led to a bilingual advantage relatively more often in younger children than they did in children between the ages of 6 and 12. Interestingly, the opposite was true for Simon-like tasks, where older children showed a bilingual advantage relatively more often. This seeming lack of convergent validity between different tasks is in line with the mixed findings in research on the subject (e.g., Ross and Melinger, 2017; Poarch and van Hell, 2019). However, it should be noted that in one study targeting the critical group (Martin-Rhee and Bialystok, 2008), a delay was inserted in two versions of the Simon Task (Simon and Rudell, 1967) leading to null results on both occasions. If we were to exclude those measures, the balance would already be slightly modified and lead to a bilingual advantage being detected 3 out of 6 times in the critical age group (i.e., a 50% detection rate as opposed to the 60% detection rate in the post-critical group). Lee et al. (2013) already reported that between the ages of 6 to 15, inhibition costs reduced rapidly on the Flanker Task whereas they remained present and relatively stable on the Simon Task. This might explain why there is a higher bilingual advantage rate for post-critical age groups on the Simon Task than on the Flanker Task; there is simply more room for a bilingual advantage to exist. Overall, null results on tasks of inhibition were more frequently reported than results of a bilingual advantage, in addition to the likely presence of a publication bias (cf. De Bruin et al., 2015), which already favors alternative over null results. Our findings therefore strengthen the pleas for caution and skepticism made by Paap et al. (2015).

In tasks measuring switching, we were able to document a nearly exclusive focus on DCCS-like tasks. Twenty-four out of 40 tasks were of this type, other tasks being employed three times or less. The results for most tasks were mixed. A bilingual advantage for switching was found relatively more often for the younger age group (63%) than for the older age group (33%). The difference was entirely driven by the results for DCCS-like tasks, on which children aged younger than 6 showed a bilingual advantage on 78% of the tasks, whereas children between the ages of 6 and 12 demonstrated one in 33% of the cases. It has been shown in the general literature on DCCS that, while at the age of 3 most children exhibit a pattern of inflexibility, by the age of 5 most children switch when they are instructed to do so (Zelazo, 2006). Our findings suggest that this switching ability arises earlier in bilingual children, resulting in a bilingual advantage during the critical period which tends to disappear in post-critical age groups. This could be influenced by the constant switches bilinguals make between their languages, as previous research has also indicated language switching to be a key determinant for bilingual advantages in CC processes (Verreyt et al., 2016).

The Opposite Worlds task (Manly et al., 2001) was applied only twice but showed a bilingual advantage on both occasions. Although the task is evidently connected to the DCCS Task, they are different in that the DCCS Task requires participants to respond to two visible cues whereas the Opposite Worlds task requires ignoring the visible cue in favor of the instruction. Furthermore, while most tasks were applied throughout childhood, the Multilocation Task (Zelazo et al., 1998) and questionnaires were only used among younger children, and the Global/Local Task (Navon, 1977) was only used with older children. For the latter, this can easily be explained as basic reading ability is required to complete the task. However, there is no clear indication as to why the Multilocation Task has never been applied with older children. Teacher/parent questionnaires seem to be used when researchers anticipate that improved CC cannot be observed in behavior yet, notwithstanding the experimental results that were gathered by Kovács and Mehler (2009) with participants as young as 7 months old. Nonetheless, we feel like the more comprehensive view these questionnaires offer might also prove useful in older age groups, that is, taking into account the possible susceptibility to parent/teacher bias.

In all, both on tasks measuring inhibition and switching, bilingual advantages were detected more frequently in the critical age group (inhibition: 56 vs. 37%; switching: 63 vs. 33%). Especially in tasks that were frequently applied, such as Stroop-like tasks, Flanker-like tasks, Go/No-go tasks, and DCCS-like tasks. There was a substantial difference in bilingual advantage detection rates, favoring the critical age group over the post-critical one. Furthermore, we found that across age groups the insertion of delay in a given task influenced outcomes greatly. Whenever response was delayed, bilingual advantages rarely emerged in the studies under scrutiny. In gift delay tasks measuring inhibition, five studies showed null results whereas only one study established a significant effect. The Multilocation Task (Zelazo et al., 1998) likewise led to null results on both occasions where it was used. Interestingly, this task required a 10-s delay before participants could answer the question at hand. These descriptive results deserve more attention in future research. Still, the most prominent finding of this review is that if young bilinguals show an advantage in EF over monolinguals, it tends to partially disappear as they grow older. Crucially, there is wide agreement in the monolingual literature that the age span from three to six is critical for CC development (Best and Miller, 2010; Chevalier et al., 2012; Lucenet and Blaye, 2014). Hence, there seems to be an overlap in the timeframe in which the bilingual advantage is mostly observed and the sensitive period for CC development. Our results are in line with what we hypothesized, namely that bilingualism might accelerate CC development, but that this is only a temporary effect and monolinguals manage to catch up at a later stage. As we already stated, similar findings are reported in literature on the relation between bilingualism and cognitive decline, providing more support for the overlap between periods of crucial CC evolution and periods in which bilingual advantages can be detected.

As a parting statement, it should be noted that the format of a systematic review restricts us to presenting descriptives, whereas an added meta-analysis could lead to more conclusive insights on the existence of cognitive advantages in EF for bilingual children. Moreover, several studies in the present review include not only native speakers, but also children who were exposed to another language slightly later on, both through high and low exposure. An analysis of the difference between these groups was beyond the scope of this review but might have influenced outcomes. However, the current review can prove extremely useful within the research field as it has shed light on terminological issues and frequently applied tasks in addition to providing a concise overview of research on the bilingual advantage in switching and inhibition in children. Moreover, it has differentiated between critical and post-critical age groups and, in doing so, was able to draw links between timeframes in which a bilingual advantage emerges and periods that are crucial for EF development in children.

## Data availability statement

The original contributions presented in the study are included in the article/supplementary material, further inquiries can be directed to the corresponding author.

## Author contributions

NP and EW conceptualized the study. NP conducted the search on Web of Science, drew up the table, and drafted the first manuscript. Both WD and EW have presented valuable feedback counting on their profound expertise in the subject at hand. All authors contributed to the article and approved the submitted version.
